# Clinical and Biological Significances of FBLN5 in Gastric Cancer

**DOI:** 10.3390/cancers15020553

**Published:** 2023-01-16

**Authors:** Xiulan Bian, Shengjie Yin, Xin Yin, Tianyi Fang, Yufei Wang, Shuo Yang, Xinju Jiang, Yingwei Xue, Fei Ye, Lei Zhang

**Affiliations:** 1 Department of Pathology, Basic Medical Science College, Harbin Medical University, Harbin 150086, China; 2 Department of Medical Oncology, Municipal Hospital of Chifeng, Chifeng 024000, China; 3 Department of Gastroenterological Surgery, Harbin Medical University Cancer Hospital, Harbin Medical University, Harbin 150086, China

**Keywords:** gastric cancer, FBLN5, fibroblast, prognosis

## Abstract

**Simple Summary:**

The purpose of this study was to investigate the effect of high expression of FBLN5 on the prognosis of gastric cancer (GC) patients. FBLN5, as a member of the fibrin family, regulates important biological processes related to cancer occurrence and development and has been proven to play an important role in a variety of cancers. However, the roles of FBLN5 in GC have not been studied to date. Therefore, we preliminarily explored the influence of FBLN5 on the progression of GC by combining biological information analysis technology and basic experiments and confirmed that FBLN5 has good application value in evaluating the prognosis of GC patients, thereby providing a reference for later in-depth study of FBLN5.

**Abstract:**

Abnormal FBLN5 expression levels are related to various cancer types. This study is the first to explore its clinical and biological significances in gastric cancer (GC). We used The Cancer Genome Atlas-GC (TCGA-GC) and Gene Expression Omnibus (GEO) databases to identify the differential expression of FBLN5, and its association with clinical pathological characteristics was analyzed. A Kaplan–Meier plotter was used to calculate the impact of FBLN5 on GC patient prognosis, and the biological functions of FBLN5 were analyzed. In addition, we constructed a GC tissue microarray, and performed an immunohistochemical staining of FBLN5 to verify our findings. Western blotting was conducted simultaneously to confirm that FBLN5 was overexpressed in GC. We found that the high level of FBLN5 mRNA in GC was associated with a poor prognosis. High FBLN5 expression levels were significantly correlated with INFc and N3 lymph node metastasis. Univariate and multivariate analyses showed that FBLN5 expression levels and lymph node metastasis rate were independent risk factors related to GC patient prognosis, which can be combined to construct a nomogram to serve patients. Therefore, we believe that FBLN5 is significantly related to the poor prognosis of GC patients. FBLN5 is a valuable prognostic indicator to evaluate the prognosis of GC.

## 1. Introduction

GC is the second most commonly occurring digestive tract malignancy and the third leading cause of cancer death worldwide [1]. The rate of early diagnosis of GC is low and about 70% of patients present with advanced GC at diagnosis [2], After the diagnosis of advanced GC, most tumor cells have invaded blood vessels or lymphatic vessels [3]. Tumor cells remain dormant and plant in distant tissues and organs; consequently, the recurrence rate of advanced GC patients with lymph node metastasis was 20% even five years after radical surgery, while the hematogenous metastasis rate was as high as 40% [4]. The tumor microenvironment (TME) of GC patients consists of diverse components and shows complex responses, and tumor cells have two-way interactions with the surrounding interstitial components. For example, fibroblasts in the TME play a role in promoting angiogenesis in tumors [5]. Fibroblasts can also activate epithelial mesenchymal transformation (EMT), RAS, and transforming growth factor-beta (TGF-β) signaling, thereby causing tumor cells to acquire interstitial morphology and lose E-cadherin. Consequently, adhesion between cancer cells is weakened [6,7] And tumor cells can easily fall off from the primary focus and spread to the blood stream, causing distant metastasis [8]. In addition, fibroblasts also regulate the infiltration, phenotype, and infiltration distribution of immune cells in the TME through a variety of factors, including chemokines (CXCL12 and CXCL16), interleukins (IL6, IL8, and IL11), and cell surface proteins (PD-1 and PD-2). These regulatory effects could weaken the immune response to the tumor and enable tumor cells to acquire metastatic capacity [9].

Although tumor-related fibroblasts have been studied using bioinformatics techniques and experiments, the roles of different fibroblast components in tumors still need to be analyzed. The fibulin (FBLN) family contains fibrin 1–7 and was widely found in the extracellular matrix (ECM), which is involved in the formation and stabilization of basement membranes, elastic fibers, and loose connective tissues [10,11]. Unlike other members of the FBLN family, FBLN5 contains a conserved RGD motif that binds to integrins and mediates endothelial cell adhesion [12,13]. As a key member, FBLN5 participates in the assembly of continuous elastin (ELN) polymers and promotes interactions between microfibers and ELN [14]. During biological processes, FBLN5 is involved in cell proliferation, the regulation of cell motility, tumorigenesis, and tissue repair [15]. FBLN5 is also a target of TGF-β in fibroblasts and endothelial cells [16,17], which affect tumor progression. Numerous studies have shown that FBLN5 acts as an inhibitor or promoter of tumor cell growth depending largely on the cancer type and environment. In individuals with ovarian cancer, FBLN5 induces cell cycle arrest and regulates the expression of cell-cycle-related proteins, thereby inhibiting the progression and metastasis of cancer cells [15]. The cancer suppressive effect is also observed in individuals with bladder cancer [18] and lung cancer [19]. In addition, FBLN5 initiates EMT and induces elevated matrix metalloenzyme expression activity to promote breast cancer cell metastasis [20]. FBLN5 also enabled the promotion of tumor metastasis in pancreatic cancer [21], cervical cancer [22], and GC. The research [23,24] on FBLN5 in GC has proved that FBLN5 is highly expressed in advanced GC and promotes the proliferation and invasion of GC cells. However, the clinical significance and biological roles of FBLN5 in GC remain unclear so far; given this, these aspects were evaluated in this study.

## 2. Materials and Methods

### 2.1. Patients and Tissue Samples

We collected tumor tissue specimens, adjacent healthy tissue specimens, and clinical data of 269 GC patients who underwent radical gastrectomy at Harbin Medical University (HMU) Cancer Hospital. These data were used to construct the HMU-GC cohort and updated in December 2021. All samples were derived after obtaining written informed consent from patients. The study was approved by the Institutional Review Committee of the Affiliated Cancer Hospital of Harbin Medical University. The data were stored in the GEO repository (GSE184336 and GSE179252). RNA isolation, library construction, and mRNA sequencing were performed by Novogene Biotech Co., Ltd. (Beijing, China).

### 2.2. Data Processing

We normalized the gene length and sequence depth of the high-throughput sequencing dataset obtained from The Cancer Genome Atlas-Stomach Adenocarcinoma (TCGA-STAD) and HMU-GC data and converted them into values expressed in terms of transcripts per kilobase million (TPM). We used the ComBat (22257669) algorithm in the “sva” package to correct the batch effects from non-biotechnological deviations. We combined the HMU-GC and TCGA-STAD databases and used the resulting cohort as our training cohort. For microarray datasets GSE15459 and GSE62254 obtained from the GEO database, we downloaded the original CEL file to calculate the absolute mRNA expression levels. This process was corrected using the ComBat (22257669) algorithm as our validation cohort.

### 2.3. Bioinformatics Analyses

In the training cohort, the patients were divided into high- and low-expression groups based on the median level of FBLN5 mRNA expression. PCA analysis performed z-score on the expression spectrum and further used the prcomp function for dimension reduction analysis to obtain the reduced matrix. Then, we used the “limma” package to analyze the differentially expressed genes between the high- and low-expression groups. The difference multiple was selected twice, *p* value < 0.05, and FDR was used. Next, we carried out gene ontology (GO) analysis. We used the GO annotation of genes in the “org. Hs. eg. db” package to display the molecular function (MF), cellular component (CC), and biological process (BP). In order to perform Kyoto Encyclopedia of Genes and Genomes (KEGG) analysis, we obtained the latest genetic annotations for the KEGG Pathway through the KEGG rest API (https://www.kegg.jp/kegg/rest/keggapi.html) and used “cluster Profiler” for enrichment analysis. KEGG (assessed on 7 April 2022). The “cluster Profiler” package was also used in gene set enrichment analysis (GSEA) to explore abundant pathways in the high-expression group, using reference genomes as a hallmark and a |normalized enrichment score (NES)| > 1, nominal (NOM) *p*-value < 0.05, and FDR *q*-value < 0.25. The PPI network was built using the Search Tool (STRING) (version 11.5) (https://string-db.org/) to search for interacting genes, and the minimum required interaction score was set to 0.9. STRING (assessed on 10 November 2021). Receiver Operating Characteristic Curve (ROC) analysis was carried out using the “pROC” package and “timeROC” package, and the analysis results were visualized with “ggplot2” package. Visualization of risk factor graph was done with “ggplot2” package. The CIBERSORT and TIMER algorithms were used to assess the relationship between the expression levels of FBLN5 and immune infiltration in each tumor sample. The ESTIMATE algorithm was used to estimate the content of immune cells and stromal cells in individuals with GC, and assessed the immune score, stromal score, and tumor purity. We downloaded the somatic mutation data of the TCGA-STAD cohort through the GDCquery_Maf() function (pipelines=“mutect2”) of the TCGA “biolinks” package and used the “maftools” package to analyze and visualize the top 20 individual cell variants with the highest mutation frequency between the high- and low-expression groups. Chemotherapy sensitivity was based on the PRISM database. We explored FBLN5 as a biomarker for drug-efficacy evaluations through a common immunotherapy public database TIDE: Tumor Immune Dysfunction and Exclusion (http://tide.dfci.harvard.edu/). TIDE (assessed on 11 June 2021). We applied the ssGSEA algorithm to calculate the EMT score for each tumor sample. Finally, we used the “rms” and “survival” package to draw the nomogram and calibration diagram, along with the “survival” package and stdca.R file to draw the decision curve analysis (DCA) diagram. We thank Sangerbox 3.0, version 1.1.3, Yongxin Liu (Shenzhen, China) for the above process analysis [25].

### 2.4. Immunohistochemistry

We selected 180 human GC tissue samples from Harbin Medical University Cancer Hospital. After performing the tissue embedding, sectioning, and HE staining processes, the histological chip was finally constructed. We placed the histological chip in an oven at 62 °C for 2 h and performed alcohol dehydration after conventional xylene dewaxing, followed by repair at a high temperature of 120 °C in EDTA buffer at a pH of 7.4 for 3 min, and allowed for cooling to occur naturally. Endogenous peroxidase activity was inhibited using 0.3% hydrogen peroxide and methanol (30 min). Rinsing was performed three times using PBS for 10 min each. Goat serum was used for blocking at room temperature for 1 h and the serum was discarded. Next, the FBLN5 rabbit polyclonal antibody (ABclonal, Woburn, MA, USA) was diluted at a ratio of 1:150, dropped on the glass slide, and incubated overnight at 4 °C (not more than 16 h). Secondary antibodies were applied at room temperature for 40 min in a moist box the next day, and the color development reaction was performed using diaminobenzidine (DAB) staining. Then, we performed dyeing and fixation with hematoxylin, and ammonia reverse blue staining. The staining results of all sections were evaluated by two professional pathologists. We scanned the TAM at 200× total magnification using the Leica pathology microscope DM4B (Leica Microsystems GmbH, Wetzlar, Germany) and assessed tumor staining using a semiquantitative immunohistochemical H-score (0–300) based on staining intensity. The scoring criteria are negative (0), weak (1), medium (2), or strong (3), and the score is multiplied by the percentage of dyed area at this intensity. The immunohistochemistry scores were divided into high expression or low expression based on survival using X-tile software.

### 2.5. Western Blotting

Collected cells (GES, AGS, BGC-823, HGC-27, and MKN-28) were lysed on ice with phosphatase and protein inhibitors in RIPA buffer for 30 min, then centrifuged at 13,000 rpm for 15 min. The cell debris was removed, the supernatant was obtained using a pipette, a certain amount of 5× loading buffer was added, and samples were heated at 100 °C for 10 min. The BCA protein analysis kit (Thermo Scientific, Waltham, MA, USA) was used to quantitatively detect the protein concentration. The protein was dissolved using 12% SDS-PAGE, the protein was transferred to a PVDF membrane at low temperatures, 5% skimmed milk powder was used for blocking for 2 h, and the membrane was incubated with the primary antibody overnight at 4 °C (FBLN5: 1:1000, ABclonal, USA; β-Tubulin: 1:1000, ABclonal, USA). The membrane was incubated for 1 h the next day with a horseradish peroxidase-labeled secondary antibody (1:5000). The ECL kit (Thermo Scientific, USA) was used to assess the expression of individual proteins. The experiment was conducted in triplicate (Figure S1 of Supplementary Materials).

### 2.6. Statistical Analyses

Statistical analyses were performed using SPSS statistical software (25.0). The Kruskal–Wallis test was used for continuous variable data, and the Chi-square test was used to analyze the correlation between the FBLN5 mRNA levels, protein levels, and clinicopathological characteristics of patients. The risk ratio (HR) and 95% confidence interval (CI) were estimated using the survival package of the Cox regression model. Survival analysis was performed using the Kaplan–Meier curve. A two-tailed *p*-value < 0.05 was considered to be statistically significant for all statistical analyses.

## 3. Results

### 3.1. The Correlations between the FBLN5 mRNA Expression Level and Prognostic, Clinicopathological Features

**Figure 1:**
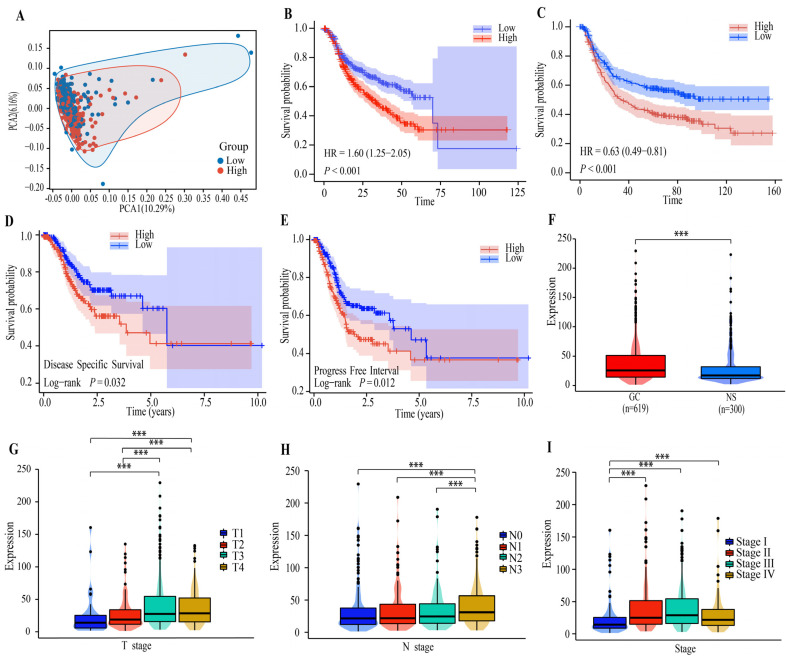
The associations between the FBLN5 mRNA expression levels and prognostic and clinicopathological features. (**A**) The PCA analysis assessed the grouping situation of patients with different levels of FBLN5 expression. (**B**) Overall survival analysis of patients with high- and low-expression levels of FBLN5 in the HMU-TCGA database (*p* < 0.001). (**C**) Overall survival analysis of patients with high- and low-expression levels of FBLN5 in the GEO database (*p* < 0.001). (**D**) DSS analysis of patients with high- and low-expression groups of FBLN5 (*p* < 0.05). (**E**) PFI analysis of patients with high- and low-expression groups of FBLN5 (*p* < 0.05). (**F**) Analysis of the differential expression of FBLN5 in GC (619 cases) and normal tissues (300 cases). (**G**–**I**) The expression of FBLN5 in GC tissues in patients with different grades of disease (*p* < 0.05). *** *p* < 0.001. (HMU, Harbin Medical University; TCGA, the Cancer Genome Atlas; GEO, gene expression omnibus; GC, gastric cancer; DSS, disease-specific survival; PFI, progress-free interval).

We took the median of FBLN5 mRNA expression levels as the cut-off value and divided the patients into an FBLN5 high-expression group and FBLN5 low-expression group. We showed the clinical baseline data sheet in Supplementary Table S1. The PCA analysis showed the intra-group and inter-group consistency between the two groups (Figure 1A). The survival of patients in the high-expression group was significantly poor. The median survival was 33.03 months, and the median survival of patients in the low-expression group was 68.37 months (*p* < 0.001, HR: 1.60, 95% confidence interval [CI]: 1.25–2.05) (Figure 1B). In addition, in the validation cohort, we found that groups exhibiting the median level of FBLN5 mRNA expression had the same prognosis, while the high-expression group also had a poor prognosis (*p* < 0.001, HR = 0.63, 95% CI: 0.49–0.81) (Figure 1C). Subsequently, we used the TCGA database to evaluate the disease-specific survival (DSS) (Figure 1D) and progress-free interval (PFI) (Figure 1E) of patients in the high- and low-expression groups of FBLN5; the results showed that the DSS and PFI of patients with high expression of FBLN5 were significantly worse. Next, we compared the expression of FBLN5 in tumor tissues and healthy tissues adjacent to the tumor in the training cohort and found that FBLN5 was expressed at relatively high levels in cancer cells (Figure 1F). In addition, we analyzed the relationship between FBLN5 mRNA expression levels and clinical features and found that FBLN5 expression levels were not affected by the sex and M stage (Figure S2 in Supplementary Materials), while FBLN5 expression levels were significantly associated with the T stage (Figure 1G) (*p* < 0.001), N stage (Figure 1H) (*p* < 0.001), and pTNM stage (*p* < 0.001) (Figure 1I).

### 3.2. Biological Functions of FBLN5

**Figure 2:**
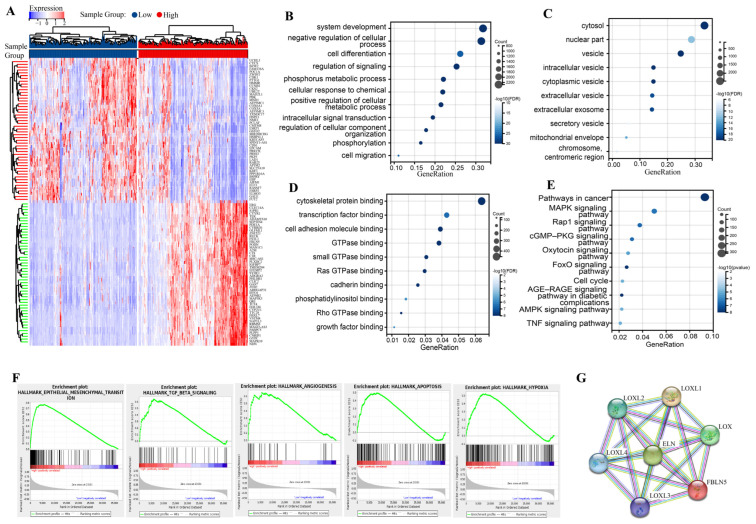
Analyses of biological functions of FBLN5. (**A**) Analysis of the differential genes between groups expressing high and low levels of FBLN5 using the limma package. Representative GO enrichment analysis between groups expressing high and low levels of FBLN5 based on TCGA-GC data, which included the following: (**B**) Biological processes (BP) associated with FBLN5 gene functions, (**C**) Cellular components (CC) of FBLN5 gene functions, (**D**) Molecular functions (MF) associated with FBLN5 gene functions. (**E**) Representative KEGG enrichment analysis between groups expressing high and low levels of FBLN5 based on TCGA-GC data. (**F**) GSEA analysis of the group expressing high levels of FBLN5 using hypoxia, angiogenesis, TGF-β, EMT, and apoptosis signals (ALL|normalized enrichment score (NES)| > 1, nominal (NOM) *p*-value < 0.05 and FDR *q*-value < 0.25). (**G**) PPI analysis of FBLN5-associated proteins.

**Figure 3:**
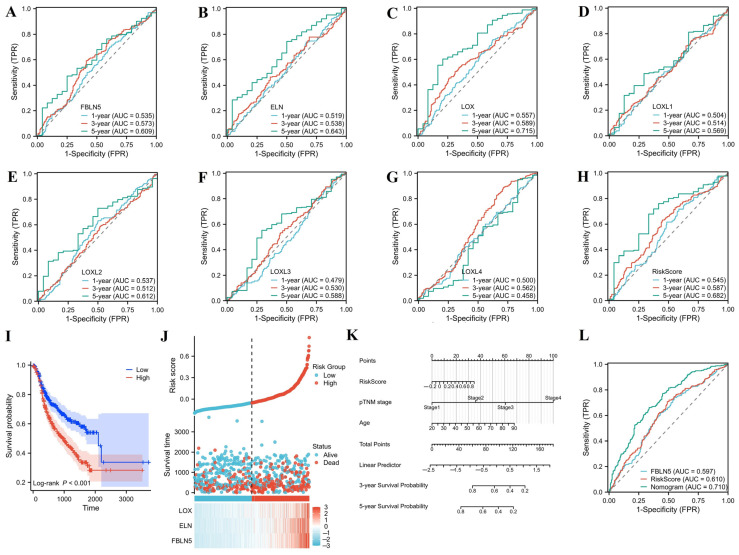
Predicting the prognosis of GC patients based on the mRNA expression levels of FBLN5. (**A**–**G**) ROCs analyzed the prognostic ability of FBLN5, ELN, LOXL1, LOXL2, LOXL3, and LOXL4 mRNA expression levels. (**H**) Prognostic risk score for FBLN5 expression, ELN expression, and LOX expression. (**I**) Assessment of the patient’s OS based on a prognostic risk score. (**J**) Risk factor graph to visualize the trend of prognostic model. (**K**) Nomogram prediction model based on risk score, pTNM stage, and age. (**L**) ROC analysis based on FBLN5 expression, risk score, and nomograph prediction model. (OS: overall survival).

Based on limma analysis, we identified 2663 upregulated genes and 5957 downregulated genes (Figure 2A) (Supplementary Table S2). We performed a GO analysis of these genes and found that FBLN5 and any related genes were involved in biological processes (BPs) such as system development, negative regulation of cellular processes, cell differentiation, regulation of signaling, phosphorus metabolic process, and cellular response to chemical stimulus (Figure 2B), and participated in the cytosol, nuclear part, vessel, intelligent vessel, and cyclomatic vessels in CCs (Figure 2C). MFs involved processes such as cytoskeletal protein binding, transcription factor binding, cell adhesion molecule binding, GTPase binding, and small GTPase binding (Figure 2D) (Supplementary Table S3). The KEGG analysis results showed that FBLN5 was mainly involved in the MAPK, Rap1, and cGMP-PKG signaling pathways (Figure 2E). (Supplementary Table S3). GSEA analysis results showed that the signaling pathways associated with EMT signaling, TGF-β signaling, apoptosis, hypoxia, and angiogenesis were significantly enriched in patients with high FBLN5 expression levels (Figure 2F) (Supplementary Table S4). We analyzed the PPI network of FBLN5 and identified interactions between FBLN5 and LOX, LOX1L, LOX2L, LOX3L, LOX4L, and ELN (Figure 2G). Furthermore, we analyzed the ability of PPI-network-related proteins to judge the prognosis, and combined this with the gene expression level and traditional clinicopathological factors to construct a prognosis risk model. First, we analyzed the ability of FBLN5, ELN, LOX, LOXL1, LOXL2, LOXL3 and LOXL4 mRNA levels to judge prognosis through ROCs (Figure 3A–G). We found that the ability of a single gene to judge the prognosis was not satisfactory. Then, we used the Cox method to evaluate the prognostic significances of each gene (Supplementary Table S5). The results showed that FBLN5, ELN, and LOX are prognostically related genes. We obtained a prognostic risk score based on the expression levels of FBLN5, ELN, and LOX. The prognostic risk score has a more stable prognostic ability than a single gene (Figure 3H). A KM survival curve analysis found that patients with higher prognostic risk scores have significantly worse rates of overall survival (OS) (Figure 3I). In addition, we built a risk factor graph to visualize the trend of prognostic models, which included the expression of FBLN5, ELN, and LOX (Figure 3J). Finally, we incorporated the prognostic risk score and traditional clinicopathological factors into the multivariate Cox risk regression (Supplementary Table S6) and established a prognostic nomograph (Figure 3K). ROC analysis showed that the nomograph constructed by combining the prognostic risk score, pTNM stage, and age can better judge the prognosis of GC patients (Figure 3L).

### 3.3. Relationships between FBLN5 and Immunity

**Figure 4:**
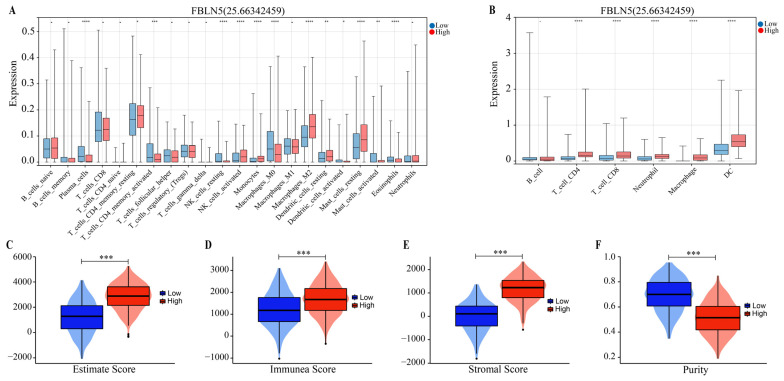
Relationships between FBLN5 and immunity. (**A**) CIBERSORT and (**B**) TIMER algorithms preliminarily predicted the relationship between the FBLN5 expression levels and immune infiltration in each tumor sample. The (**C**) ESTIMATE algorithm estimated the content of immune and stromal cells in GC, and helped to predict the (**D**) immune score, (**E**) stromal score, and (**F**) tumor purity. * *p* < 0.05, ** *p* < 0.01, *** *p* < 0.001, **** *p* < 0.0001.

We analyzed the relationships between the expression levels of FBLN5 and the tumor immune microenvironment. In Figure 4A, we showed that the expression of FBLN5 was significantly correlated with the infiltration of CD4+ T cells, NK cells, M0 macrophages, M2 macrophages, mast cells, and other immune cells using CIBSCORT analysis (ALL *p* < 0.05). A TIMER analysis showed that the levels of CD4+ T cells, CD8+ T cells, neutrophils, macrophages, and dendritic cells were statistically significant in the group expressing high levels of FBLN5 (Figure 4B) (ALL *p* < 0.05). In addition, the high-expression group had a higher estimate score (Figure 4C), immune score (Figure 4D), stromal score (Figure 4E), and lower tumor purity (Figure 4F) (ALL *p* < 0.05), which indicate that FBLN5 has a significant effect on the immune status of the tumor microenvironment.

### 3.4. Association between FBLN5 and Tumor Progression

**Figure 5:**
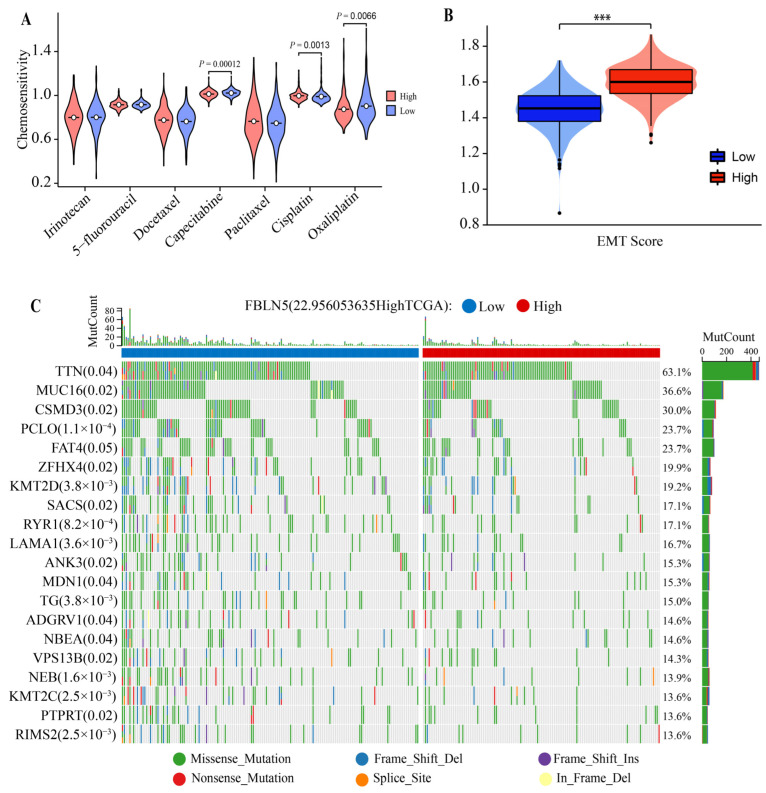
Relationships between FBLN5 and tumor progression. (**A**) Chemotherapy analysis based on FBLN5 expression levels. (**B**) EMT score. (**C**) Mutations in FBLN5 during the regulation of cancer progression. *** *p* < 0.001.

We evaluated the sensitivity of GC patients to irinotecan, 5-fluorouracil, docetaxel, capecitabine, paclitaxel, cisplatin, and oxaliplatin, based on FBLN5 expression levels, and found that the expression levels of FBLN5 affected the drug sensitivity of capecitabine, cisplatin, and oxaliplatin (Supplementary Table S7) (Figure 5A). At the same time, we evaluated the value of FBLN5 to assess the sensitivity of immunotherapy checkpoints (Table 1). In addition, we found that patients in the high-expression group had higher EMT scores compared to the low-expression group (Figure 5B), which may indicate that high FBLN5 expression levels might promote the isolation of GC cells from the lesion and their transfer to other sites through the EMT process, which confirmed that FBLN5 was closely associated with tumor progression. In addition, we performed exon missense mutation analysis (Figure 5C) in the high- and low-expression groups based on an analysis of data from TCGA-STAD. The results showed that the FBLN5 mutation rate in the high expression group was low, i.e., FBLN5 enabled the stable regulation of patients with advanced GC, which was closely related to disease progression in GC patients.

### 3.5. Expression of FBLN5 and Patient Prognosis

**Figure 6:**
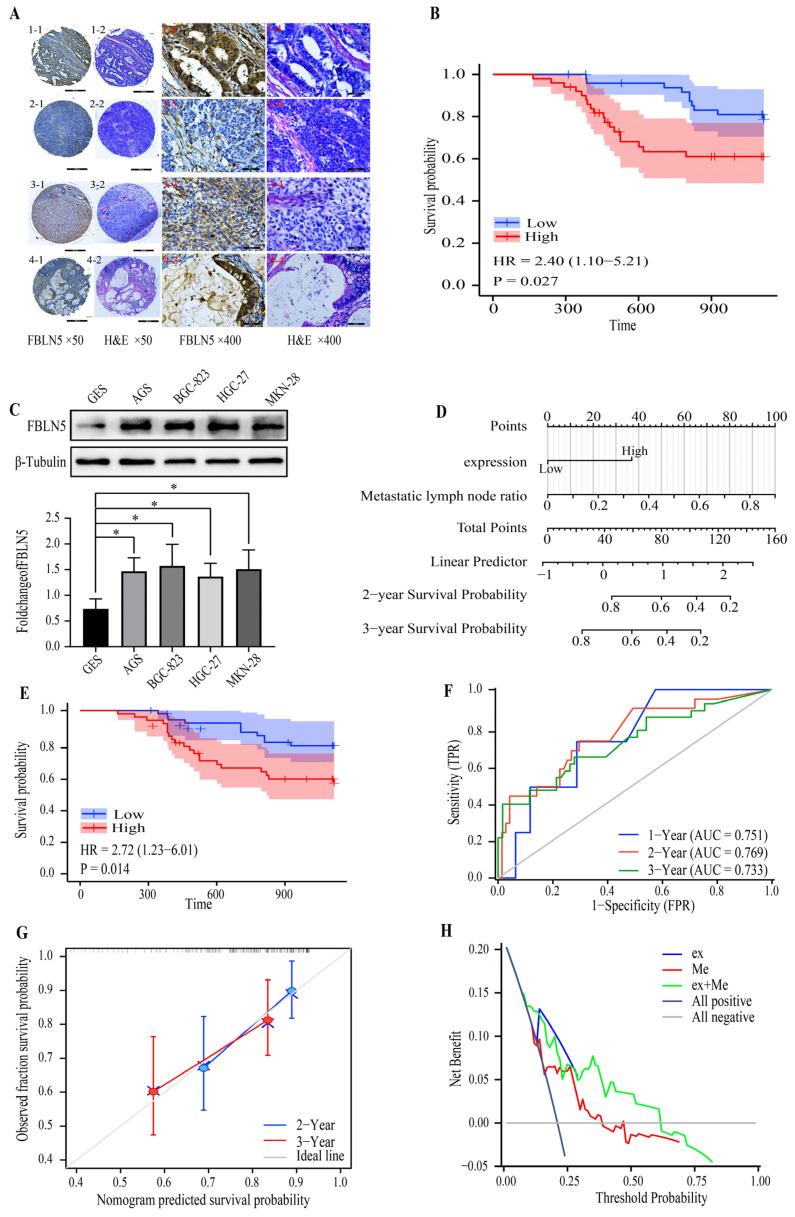
FBLN5 expression levels and prognostic model establishment. (**A**) FBLN5 immunohistochemistry and H&E staining of GC TMA, ×50 and ×400 total magnification. (**A1**) Well-differentiated adenocarcinoma. High expression of FBLN5 in the cytoplasm of cancer cells and tumor interstitial fiber cells. (**A2**) Poorly differentiated adenocarcinoma. FBLN5 is expressed at low levels in the cytoplasm and highly expressed in interstitial fibroblasts. (**A3**) Another poorly differentiated adenocarcinoma. FBLN5 is highly expressed. (**A4**) Mucinous adenocarcinoma. FBLN5 is highly expressed in the cytoplasm of mucinous adenocarcinoma and interstitial fibroblasts. (**B**) KM survival analysis curve of patients with different FBLN5 expression levels in a tissue micro-array. (**C**) FBLN5 expression levels in different cell lines ± s.d (no. of replicates = 3). (**D**) A nomograph prediction model based on the expression levels of FBLN5 and lymph node metastasis rate. (**E**) Survival curve based on the nomogram prediction model. (**F**) ROC curve predicted the feasibility of the nomograph prediction model. (**G**) Two-year and three-year calibration analysis. (**H**) DCA diagram based on the expression of FBLN5 and metastasis. * *p* < 0.05.

In order to verify the effect of FBLN5 on GC prognosis, we performed IHC staining of FBLN5 using the histological chip (Figure 6A), and the staining results showed that FBLN5 was mainly expressed in the cytoplasm of cancer cells and interstitial fibrous cells of tumors. At the same time, we found that there are differences in the location of FBLN5 between the high-expression group and the low-expression group. In well-differentiated adenocarcinoma, FBLN5 was highly expressed in the cytoplasm of cancer cells and tumor interstitial fiber cells. FBLN5 was expressed at low levels in the cytoplasm of poorly differentiated adenocarcinoma cells and highly expressed in interstitial fiber cells. However, FBLN5 was highly expressed in another poorly differentiated adenocarcinoma, suggesting that there is heterogeneity in the expression levels of FBLN5 in tumor cells with the same degree of differentiation. In addition, in mucinous adenocarcinoma, FBLN5 is highly expressed in the cytoplasm of cancer cells and interstitial fibroblasts. We then evaluated the results of IHC staining and found that overall survival (OS) was significantly higher in the low-expression group than in the high-expression group (*p* < 0.05, HR = 2.40, 95% CI: 1.10–5.21) (Figure 6B). At the same time, we also measured the expression levels of FBLN5 in a healthy gastric epithelial cell line and several GC cell lines (Figure 6C) and found that FBLN5 was expressed at significantly higher levels in GC cell lines, suggesting that FBLN5 expression levels may be related to the histological type of GC tissues. To further evaluate the relationship between FBLN5 expression levels and patient prognosis, we performed univariate and multivariate Cox analyses (Table 2) and found that FBLN5 expression and lymph node metastasis rates were independently correlated with patient prognosis (*p* < 0.05). In addition, after Chi-square analysis, we found that the expression level of FBLN5 was related to the tumor infiltration pattern and N stage. Patients with high FBLN5 expression levels were more inclined to exhibit the INFc tumor infiltration pattern and N3 stage (Table 3). We then established a prognostic nomogram based on the results of multivariate Cox analysis (Figure 6D). After calculating the scores of each patient using the nomogram, patients could be grouped according to the median value, and the survival period of patients in the high-risk group was shorter (Figure 6E). The area under the time-dependent ROC curve of one-year, two-year, and three-year prognoses were 0.751 (0.564–0.938), 0.769 (0.647–0.891), and 0.733 (0.612–0.855), respectively (Figure 6F). In addition, the C-index was 0.705 (0.659–0.752) during calibration (Figure 6G) and the decision curve analysis (DCA) (Figure 6H), which could be more reflective of patient prognosis. Moreover, the DCA diagram also clearly showed that the combination of FBLN5 expression and lymph node metastasis rate to assess patient prognosis had better application prospects.

### 3.6. FBLN5 in Other Cancers

**Figure 7:**
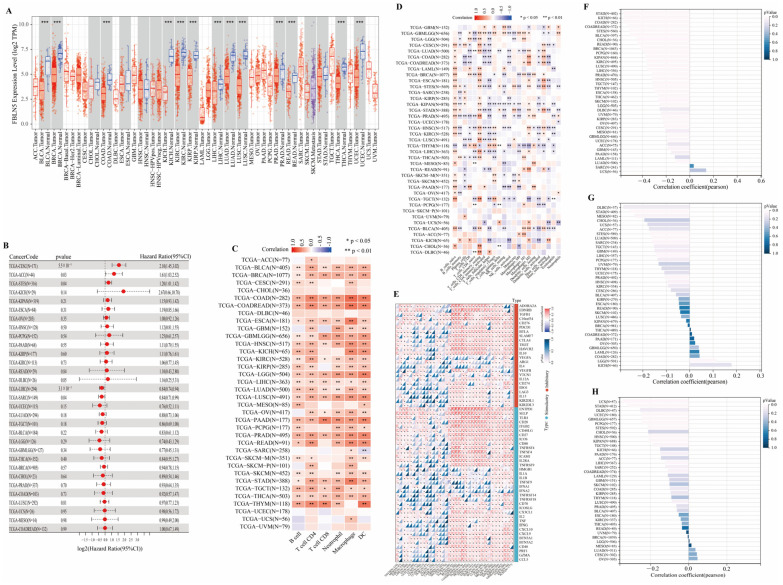
Application of FBLN5 in other cancers. (**A**) Expression levels of FBLN5 in various cancers. (**B**) Prognostic analysis of FBLN5 in various cancers. (**C**) TIMER algorithm and (**D**) CIBERSORT algorithm preliminarily predicted the relationship between the expression level of FBLN5 and immune infiltration in various types of tumors. (**E**) Pan-cancer analysis of FBLN5-related immune checkpoints, along with (**F**) tumor purity, (**G**) TMB, and (**H**) MSI analyses to evaluate the prospect of using FBLN5 for targeted pan-cancer treatment. * *p* < 0.05, ** *p* < 0.01, *** *p* < 0.001.

The above results suggest that FBLN5 could be used as a good indicator of GC patient prognosis. We further explored whether FBLN5 could also be used as a prognostic indicator in other cancers using a combination of the expression levels of FBLN5 in various cancer types and their prognosis. The analysis results showed that FBLN5 levels were also indicative for the prognosis of hepatocellular carcinoma, and the application prospects of FBLN5 in hepatocellular carcinoma are expected to be further explored in clinical practice (Figure 7A,B). We simultaneously evaluated FBLN5 expression levels and immune cell invasion in various types of cancer using TIMER and CIBSCORE analyses. The TIMER analysis showed that FBLN5 was positively correlated with immunity in most tumors (Figure 7C). However, the CIBSCORE analysis showed that FBLN5 showed a negative correlation with immune response in most tumors (Figure 7D). Hence, the immune invasion caused by FBLN5 in various tumors needs to be investigated further. In addition, an analysis of immune checkpoints showed that FBLN5 levels were positively correlated with immune checkpoints and most carcinomas, such as lung adenocarcinoma (STES), transitional cell carcinoma of the bladder (BLCA), and lung squamous cell carcinoma (LUSC), which could guide effective immunotherapy for these tumors (Figure 7E). Finally, we studied the pan-cancer application prospects of FBLN5 in targeted therapy and performed a tumor purity analysis (Figure 7F), TMB analysis (Figure 7G), and MSI analysis (Figure 7H), and found that the expression of FBLN5 showed a trend of negative correlations with TMB and MSI, which proved that targeted therapy was not supported when FBLN5 expression was high. FBLN5 has cancer-promoting effects in some tumors, cancer-suppressive effects in some tumors, and supports targeted therapy in some tumors while supporting immunotherapy in some tumors. Hence, the mechanisms of action of FBLN5 in various tumors need to be analyzed according to different tumor types.

## 4. Discussion

GC is a kind of highly malignant tumor of the digestive tract that exhibits a complex tumor microenvironment. In this study, we used TCGA-HMU GC data to explore the biological functions of FBLN5 in GC. We determined the FBLN5 mRNA and protein levels and analyzed the relationship between the expression levels of FBLN5 and clinicopathological characteristics and patient prognosis. We found that FBLN5 not only played an important role in the tumor microenvironment, but also served as a potential marker for tumor-related fibroblastogenic prognosis.

Fibrins are a family of seven extracellular matrix proteins, including fibulin-1, fibulin-2, fibulin-3, fibulin-4, fibulin-5, fibulin-6, and fibulin-7. Fibrins are involved in complex biological processes such as cell adhesion, migration, or proliferation, and are widely distributed and often associated with the vascular system and elastic tissue. Different members of the fibrin family are expressed in both tumor and mesenchymal cells, and affect tumor progression [26]. FBLN5, as a fibroblast-derived extracellular matrix protein, contains Arg-Gly-Asp (RGD) motifs and calcium-bound EGF-like domains. FBLN5 can promote endothelial cell adhesion through interactions between integrin and RGD motifs. FBLN5 is not only essential for the formation of elastic fibers [14], but also plays a role in vascular development and remodeling [12] as a vascular ligand for integrin receptors. FBLN5 has been shown to bind to TGF-β in fibroblasts and endothelial cells [16,17]. TGF-β initiated pathological carcinogenic EMT processes during fibrosis and tumor formation [27,28], which confirmed that FBLN5 might play an important role in tumor progression. In recent years, the number of studies on the roles of FBLN5 in tumor processes have increased significantly. Yong-Hun et al. [29] found that FBLN5 initiated EMT through a matrix metalloproteinase-dependent mechanism and enhanced the extent of EMT induced by TGF-β-induced breast epithelial cells, suggesting that FBLN5 had tumor-promoting functions in breast cancer. In addition, a study by Mary et al. [21] showed that FBLN5 blocked the interaction between fibronectin and integrins, thereby directly limiting the generation of reactive oxygen species driven by the ECM and promoting pancreatic cancer progression. However, the significance of FBLN5 in GC has not been studied to date. Hence, we want to explore the clinical significance of FBLN5 in GC by further using the TCGA-HMU GC database and histological chips.

In our study, after PCA analysis, we found that the scatter points corresponding to the two groups of samples showed mutual aggregation in the group, indicating that the repeatability within the group is relatively good and the sample data are very similar; however, the difference between the groups is relatively weak. We analyzed that the reason for the low discrimination between groups might be that the expression levels of FBLN5 in high- and low-expression groups is a continuous variable, resulting in a small gap between two groups. It was found that the high expression level of FBLN5 was significantly related to the T stage, N stage, and pTNM stage. The FBLN5 expression level in patients showed an increasing trend with an increase in tumor progression, lymph node metastasis, and pTNM stage, and the patient prognosis was poor, which suggests that FBLN5 might be a biomarker of tumor progression. The study conducted by Mauricio et al. [30] confirmed that the abundance of tumor associated fibroblasts (CAFs) was often associated with prognostic parameters of malignant oral squamous cell carcinoma. These prognostic parameters include the pTNM stage, tumor grade, recurrence, and depth of invasion. Thus, the expression of CAFs increased with an increase in the stage of the disease in patients. FBLN5, as a member of the fibrin family, could explain the phenomenon that the expression of FBLN5 increased significantly with an increase in the stage in GC patients. KEGG pathway analysis showed that FBLN5 was mainly enriched in the MAPK signaling pathway. Yi et al. [31] showed that ubiquitin binding enzyme E2T (UBE2T) knockout inhibited the progression of lung adenocarcinoma by targeting FBLN5. The increase in UBE2T knockout will result in the expression of FBLN5 and inhibit the activation of p-ERK, p-GSK3β, and β-catenin, which indicates that FBLN5 may regulate the MAPK/ERK signaling pathway. In addition, it was confirmed [32] that LOXL1, the protein interacting with FBLN5, stimulated angiogenesis through the LOXL1-FBLN5/avb3 integrin/FAK-MAPK axis in intrahepatic cholangiocarcinoma, which resulted in favorable conditions for cancer cell metastasis. This suggests that FBLN5 was closely related to the RGD motif-integrin/MAPK axis during the process of promoting cancer cell metastasis. We simultaneously found, through GSEA analysis, that FBLN5 was significantly enriched in the pathways associated with TGF-β signaling, EMT signaling, apoptosis, hypoxia, and angiogenesis. This showed that FBLN5 was closely related to the tumor microenvironment and could promote tumor metastasis through various methods. In breast cancer, FBLN5 initiated EMT and enhanced the process of TGF-β-induced EMT [20]. In addition, hypoxia and TGF-β synergistically induced high FBLN5 expression levels in pancreatic cancer. This was associated with a poor prognosis in pancreatic cancer patients [21]. Kazuhiro et al. showed that FBLN5 was directly related to angiogenesis [33], which fully confirmed that the poor prognosis of GC patients caused by FBLN5 overexpression was closely related to hypoxia, angiogenesis, MAPK signaling pathway, EMT signaling pathway, and TGF-β activation, suggesting that FBLN5 could be an important indicator of the poor prognosis of GC. Through the PPI network map, we found that FBLN5 interacts with ELN. Our team’s previous research found that the mRNA expression of ELN was positively correlated with the molecular markers of fibroblasts, especially VIM; it might be a useful prognostic indicator for predicting GC prognosis by regulating EMT [34], which might indicate that fibroblasts could also be regulated by the expression of FBLN5.

Richard et al. [9] referred to CAFs and the ECM as “stromal”, which could effectively inhibit the activity of immune cells and participate in tumorigenesis, progression, metastasis, and treatment resistance. As FBLN5 is an extracellular matrix protein derived from fibroblasts, we suspect that FBLN5 also inhibited the activity of immune cells and allowed for the immune escape of cancer cells to occur. CIBSCORE analysis showed that the expression of M2 macrophages and dendritic cells was significantly increased in the FBLN5 overexpression group. The high expression of FBLN5 might be closely related to the infiltration of immunosuppressive cells, such as CAFs, tumor-associated macrophages (TAMs), and dendritic cells. M2 macrophages are immunosuppressive cells that were related to the high expression levels of IL-10, VEGF, and matrix metalloproteinase (MMP). They express a large number of scavenger receptors, and have functions such as the promotion of angiogenesis, tissue reconstruction, damage repair, and tumor genesis and development. They have immunosuppressive effects on the development of cancer cells [35,36]. Furthermore, tumor-related dendritic cells produce related factors that induce the proliferation and angiogenesis of regulatory T cells and promote the immunosuppression of the microenvironment. In addition, patients with high FBLN5 expression levels had higher immune scores, stromal scores, and ESTIMATE scores, ans well as lower tumor purity. Hence, we hypothesize that patients with high FBLN5 expression levels may exhibit more fibroblasts that play an immunosuppressive role. Hence, it was essential to study its relationship with the immune microenvironment further.

In clinical settings, Chi-square analysis showed that the FBLN5 expression levels were significantly associated with the INFc and N3 stage. Zhao et al. [37] confirmed that there was an association between the INFc tumor infiltration pattern and the deep invasion, immunosuppression, and poorly differentiated phenotype of tumors, which indicates that patients would receive a poor prognosis. The prognosis of patients also worsened with an increase in lymph node metastasis. Therefore, high FBLN5 expression levels might be related to advanced GC. Cox univariate and multivariate analyses showed that the expression levels of FBLN5 and lymph node metastasis rate were independent risk factors related to the prognosis of GC patients. In our analysis, there are two possible reasons that might be responsible for the relatively lower number of independent prognostic risk factors. First, the number of patients was low; we selected 180 patients in total, but only 100 of them survived for an adequate period. Secondly, the survival period of patients with GC is relatively low. Hence, we should attempt to extend the survival time of patients and ensure that they are followed up with regularly. Based on the results of the Cox univariate and multivariate analyses, we used the combination of the FBLN5 expression level and lymph node metastasis rate to build a nomogram prediction model for evaluating the prognosis of patients. We found that the C-index was 0.705 (0.659–0.752). The AUC was 0.751 (0.564–0.938) in one year, 0.769 (0.647–0.891) in two years, and 0.733 (0.612–0.855) in three years. Therefore, in summary, FBLN5 is potentially valuable in both clinical and basic research.

Finally, we explored the roles of FBLN5 in evaluating other cancers through bioinformatics and our results showed that FBLN5 had good application prospects for assessment of the prognosis of patients with hepatocellular carcinoma. Jia et al. [38] confirmed that a low FBLN5 expression level was an important indicator of a low survival rate. FBLN5 inhibited the movement of hepatocellular carcinoma through an integrin-dependent mechanism. The RGD-dependent inhibition of MMP-7 by FBLN5 may contribute to the development of new therapeutic strategies against hepatocellular carcinoma. In addition, the expression levels of FBLN5 in cancers such as STES, BLCA, and LUSC could provide guidance regarding whether patients should undergo immunotherapy or targeted therapy, the provision of personalized treatment plans to patients, and improvements in the patient survival rate and quality of life.

Several limitations exist in our study. The gene expression data and IHC data in this study came from two cohorts, and it is difficult to integrate and analyze omics data at different molecular levels to achieve mutual validation. Secondly, the number of patients we included in the study was relatively small. Third, cellular experiments are essential for the discovery of prognostic biomarkers; we only verified the effect of FBLN5 expression on proliferation, migration, and invasion from the perspective of bioinformatics.

## 5. Conclusions

In conclusion, we found that the expression levels of FBLN5 in GC was significantly higher than that in healthy tissues adjacent to cancerous tissues, and high FBLN5 mRNA and protein expression levels were associated with a poor prognosis. In addition, patients with high FBLN5 expression levels were associated with INFc and lymph node metastasis. The FBLN5 expression level and lymph node metastasis rate were independent prognostic risk factors for GC patients and could be used to construct a nomogram for assessing patient prognosis. Therefore, FBLN5 was a good prognostic biomarker of GC.

## Figures and Tables

**Table 1:** The values of FBLN5 to assess the sensitivity of immunotherapy checkpoints (TIDE).

Study	Cancer Type	Treatment	Pos/Neg Cases	AUC of			
				FBLN5	CD274	CD8	TMB
Zhao 2019	Glioblastoma	PD1_Pre	8/7	0.38	0.68	0.50	n/a
		PD1_Post	6/3	0.17	0.61	0.67	n/a
VanAllen 2015	Melanoma	CTLA4	19/23	0.48	0.64	0.70	0.67
Uppaluri 2020	HNSC	PD1_Pre	8/15	0.37	0.69	0.58	n/a
		PD1_Post	9/13	0.23	0.70	0.48	n/a
Ruppin 2021	NSCLC	PD1	7/15	0.59	0.70	0.75	n/a
Riaz 2017	Melanoma	PD1_Prog	4/22	0.80	0.52	0.91	0.57
		PD1_Naive	6/19	0.54	0.27	0.43	0.62
Prat 2017	NSCLC/HNSC/Melanoma	PD1	21/12	n/a	0.58	0.56	n/a
Nathanson 2017	Melanoma	CTLA4_Pre	4/5	0.15	0.66	0.50	n/a
		CTLA4_Post	4/11	0.57	0.66	0.77	n/a
Miao 2018	Kidney	ICB	20/13	0.67	0.42	0.47	0.65
McDermott 2018	Kidney	PD-L1	20/61	0.60	0.62	0.66	0.54
Mariathasan 2018	Bladder_mUC	PD-L1	68/230	0.42	0.58	0.60	0.78
Liu 2019	Melanoma	PD1_Prog	16/31	0.56	0.56	0.58	n/a
		PD1_Naive	33/41	0.39	0.51	0.47	n/a
Lauss 2017	Melanoma	ACT	10/15	0.69	0.78	0.71	0.76
Kim 2018	Gastric	PD1	12/33	0.19	0.88	0.80	n/a
Hugo 2016	Melanoma	PD1	14/12	0.28	0.60	0.49	0.68
Hee 2020	NSCLC_Oncomine	PD1	9/12	n/a	0.45	0.56	n/a
Gide 2019	Melanoma	PD1	19/22	0.47	0.88	0.86	n/a
		PD1 + CTLA4	21/11	0.52	0.79	0.74	n/a
Chen 2016	Melanoma	PD1_Prog	6/9	n/a	0.54	0.61	n/a
		CTLA4	5/11	n/a	0.42	0.67	n/a
Braun 2020	Kidney	PD1	201/94	0.58	0.56	0.60	0.56

TIDE, tumor immune dysfunction, and exclusion.

**Table 2:** Cox univariate and multivariate analyses of FBLN5 gene expression.

Characteristics	Total (N)	Univariate Analysis		Multivariate Analysis	
		Hazard Ratio (95% CI)	*p* Value	Hazard Ratio (95% CI)	*p* Value
FBLN5 expression	100				
Low	50	Reference			
High	50	2.396 (1.102–5.205)	0.027	2.558 (1.162–5.632)	0.020
Sex	100				
Male	72	Reference			
Female	28	0.851 (0.362–2.002)	0.712		
Age	100	0.992 (0.957–1.028)	0.646		
BMI	100	0.944 (0.845–1.054)	0.303		
Tumor infiltration pattern	100				
INFa	20	Reference			
INFb	16	1.484 (0.371–5.937)	0.577		
INFc	48	1.761 (0.584–5.311)	0.315		
N/A	16	1.823 (0.489–6.790)	0.371		
Lymphatic infiltration	100				
Negative	55	Reference			
Positive	45	0.940 (0.445–1.988)	0.872		
Venous infiltration	100				
Negative	70	Reference			
Positive	30	0.592 (0.240–1.460)	0.255		
Nerve infiltration	100				
Negative	25	Reference			
Positive	75	2.243 (0.778–6.471)	0.135		
T stage	100				
T1	4	Reference			
T2	13	0.595 (0.054–6.565)	0.672		
T3	45	1.009 (0.130–7.821)	0.993		
T4	38	1.709 (0.225–12.999)	0.605		
pTNM stage	100				
I	10	Reference			
II	32	2.155 (0.259–17.902)	0.477		
III	58	4.558 (0.613–33.907)	0.138		
Metastatic lymph node ratio	100	14.056 (3.348–59.004)	<0.001	7.133 (1.241–41.011)	0.028
Borrmann type	100				
I	7	Reference			
II	19	0.291 (0.041–2.068)	0.217		
III	68	1.003 (0.236–4.270)	0.997		
IV	6	1.214 (0.171–8.621)	0.847		
Post-operative chemotherapy	100				
Without	97	Reference			
With	3	1.211 (0.164–8.915)	0.851		
Tumor location	100				
Lower third	54	Reference			
Middle and Upper third	42	1.866 (0.847–4.113)	0.122	1.589 (0.676–3.734)	0.289
Entire stomach	4	7.426 (2.017–27.337)	0.003	2.869 (0.565–14.569)	0.204
Histological type	100				
Well to moderately differentiated	46	Reference			
Poorly differentiated	26	0.592 (0.215–1.629)	0.310		
Signet ring cell	20	1.126 (0.459–2.764)	0.795		
Mucinous	8	0.323 (0.043–2.447)	0.274		
HER2 expression	100				
Positive	18	Reference			
Negative	82	0.602 (0.256–1.418)	0.246		
CEA	100				
≤5 ng/mL	86	Reference			
>5 ng/mL	14	0.679 (0.205–2.250)	0.526		
CA-199	100				
≤37 U/mL	88	Reference			
>37 U/mL	12	1.745 (0.663–4.593)	0.260		
CA724	100				
≤6 U/mL	74	Reference			
>6 U/mL	26	1.096 (0.483–2.490)	0.826		
FBLN5	619	1.004 (1.001–1.007)	<0.01	1.003 (0.998–1.007)	0.235
ELN	619	1.002 (1.000–1.003)	<0.05	1.000 (0.998–1.003)	0.698
LOX	619	1.005 (1.001–1.009)	<0.05	1.003 (0.998–1.008)	0.197
LOXL1	619	1.004 (0.999–1.009)	0.096		
LOXL2	619	1.002 (0.997–1.006)	0.499		
LOXL3	619	1.030 (0.992–1.069)	0.121		
LOXL4	619	1.012 (0.999–1.025)	0.062		<0.05
RiskScore	619	2.718 (1.444–5.116)	<0.01	2.226 (1.139–4.353)	
pTNM stage	605		<0.001		
Stage 1	81	Reference		Reference	<0.01
Stage 2	160	2.691 (1.395–5.191)	<0.01	2.491 (1.287–4.823)	<0.001
Stage 3	312	4.854 (2.633–8.947)	<0.001	4.645 (2.510–8.596)	<0.001
Stage 4	52	10.611 (5.412–20.806)	<0.001	12.565 (6.368–24.793)	
Gender	619		0.371		
Female	221	Reference			
Male	398	1.123 (0.870–1.449)	0.373		
Age	616	1.017 (1.007–1.028)	<0.01	1.025 (1.013–1.036)	<0.001

BMI: body mass index. Tumor location, tumor infiltration pattern, venous infiltration, and nerve infiltration were according to the post-operative pathology report. INFa: expanding growth and a distinct border with the surrounding tissue, INFc: infiltrating growth and an indistinct border with the surrounding tissue, INFb: in-between INFa and INFc. CEA: carcinoembryonic antigen, CA19-9: carbohydrate antigen 19-9, CA72-4: carbohydrate antigen 72-4. CEA, CA19-9, and CA72-4 were according to the tumor marker examination. Histological type, Borrmann type and pTNM stage were according to the 8th AJCC system.

**Table 3:** Relationship between FBLN5 mRNA expression and clinical features of GC patients.

Characteristic	High Expression	Low Expression	*p*
n	116	64	
Sex, n (%)			0.066
Female	24 (13.3%)	22 (12.2%)	
Male	92 (51.1%)	42 (23.3%)	
Age, n (%)			0.173
<60	59 (32.8%)	25 (13.9%)	
≥60	57 (31.7%)	39 (21.7%)	
BMI, n (%)			0.355
<24	80 (44.4%)	39 (21.7%)	
≥24	36 (20%)	25 (13.9%)	
Tumor infiltration pattern, n (%)			0.037
INFa	19 (10.6%)	17 (9.4%)	
INFb	36 (20%)	8 (4.4%)	
INFc	41 (22.8%)	27 (15%)	
N/A	20 (11.1%)	12 (6.7%)	
Lymphatic infiltration, n (%)			0.050
Negative	59 (32.8%)	43 (23.9%)	
Positive	57 (31.7%)	21 (11.7%)	
Venous infiltration, n (%)			0.209
Negative	81 (45%)	51 (28.3%)	
Positive	35 (19.4%)	13 (7.2%)	
Nerve infiltration, n (%)			0.179
Negative	26 (14.4%)	21 (11.7%)	
Positive	90 (50%)	43 (23.9%)	
T stage, n (%)			0.241
T1	6 (3.3%)	4 (2.2%)	
T2	14 (7.8%)	13 (7.2%)	
T3	42 (23.3%)	26 (14.4%)	
T4	54 (30%)	21 (11.7%)	
N stage, n (%)			0.046
N0	25 (13.9%)	25 (13.9%)	
N1	24 (13.3%)	12 (6.7%)	
N2	32 (17.8%)	9 (5%)	
N3	35 (19.4%)	18 (10%)	
pTNM stage, n (%)			0.065
I	10 (5.6%)	13 (7.2%)	
II	36 (20%)	20 (11.1%)	
III	70 (38.9%)	31 (17.2%)	
Metastatic lymph node ratio, n (%)			0.510
<0.3	87 (48.3%)	53 (29.4%)	
≥0.6	9 (5%)	3 (1.7%)	
0.3≥, <0.6	20 (11.1%)	8 (4.4%)	
Borrmann type, n (%)			0.187
1	8 (4.4%)	7 (3.9%)	
2	32 (17.8%)	17 (9.4%)	
3	62 (34.4%)	38 (21.1%)	
4	14 (7.8%)	2 (1.1%)	
Post-operative chemotherapy, n (%)			1.000
With	3 (1.7%)	1 (0.6%)	
Without	113 (62.8%)	63 (35%)	
Tumor location, n (%)			0.780
Entire stomach	4 (2.2%)	2 (1.1%)	
Lower third	65 (36.1%)	32 (17.8%)	
Middle and Upper third	47 (26.1%)	30 (16.7%)	
Histological type, n (%)			0.079
Mucinous	13 (7.2%)	5 (2.8%)	
Poorly differentiated	33 (18.3%)	11 (6.1%)	
Signet ring cell	26 (14.4%)	11 (6.1%)	
Well to moderately differentiated	44 (24.4%)	37 (20.6%)	
HER2 expression, n (%)			1.000
Negative	100 (55.6%)	55 (30.6%)	
Positive	16 (8.9%)	9 (5%)	
CEA, n (%)			0.434
>5 ng/mL	17 (9.4%)	6 (3.3%)	
≤5 ng/mL	99 (55%)	58 (32.2%)	
CA-199, n (%)			0.114
>37 U/mL	18 (10%)	4 (2.2%)	
≤37 U/mL	98 (54.4%)	60 (33.3%)	
CA724, n (%)			1.000
>6 U/mL	30 (16.7%)	17 (9.4%)	
≤6 U/mL	86 (47.8%)	47 (26.1%)	

BMI: body mass index. Tumor location, tumor infiltration pattern, venous infiltration, and nerve infiltration were according to the post-operative pathology report. INFa: expanding growth and a distinct border with the surroundinsg tissue, INFc: infiltrating growth and an indistinct border with the surrounding tissue, INFb: in-between INFa and INFc. CEA: carcinoembryonic antigen, CA19-9: carbohydrate antigen 19-9, CA72-4: carbohydrate antigen 72-4. CEA, CA19-9, and CA72-4 were according to the tumor marker examination. Histological type, Borrmann type and pTNM stage were according to the 8th AJCC system.

## Data Availability

Patients’ data were saved in the Gastric Cancer Information Management System v1.2 of Harbin Medical University Cancer Hospital (Copyright No.2013SR087424, http:www.sgihmu.com).

## Supplementary Materials

The following supporting information can be downloaded at: https://www.mdpi.com/article/10.3390/cancers15020553/s1, Figure S1: Western blot figures of FBLN5. The molecular weight range of the protein pre dye Marker we used is 10–180 kDa, which is 180 kDa, 130 kDa, 100 kDa, 70 kDa, 55 kDa, 40 kDa, 35 kDa, 25 kDa, 15 kDa and 10 kDa respectively; Figure S2: Correlation analysis of the expression level of FBLN5 mRNA with sex and M stage. (A) Correlation analysis between FBLN5 mRNA expression levels and sex (*p* > 0.05) (B) Correlation analysis between FBLN5 mRNA expression levels and M stage (*p* > 0.05); Table S1: Clinical baseline data sheet; Table S2: Variation analysis; Table S3: GO and KEGG analysis; Table S4: GSEA analysis; Table S5: Cox multivariate analysis; Table S6: Multivariate Cox risk regression; Table S7: Drug sensitivity analysis.
