# Supplementary material for: Clinical and Biological Significances of FBLN5 in Gastric Cancer
Source: Cancers (Basel). 2023 Jan 16;15(2):553. doi: 10.3390/cancers15020553 (PMC9856449; doi:10.3390/cancers15020553)
Supplement: Supplementary file 1 [file cancers-15-00553-s001.zip › cancers-2109226-supplementary.pdf]

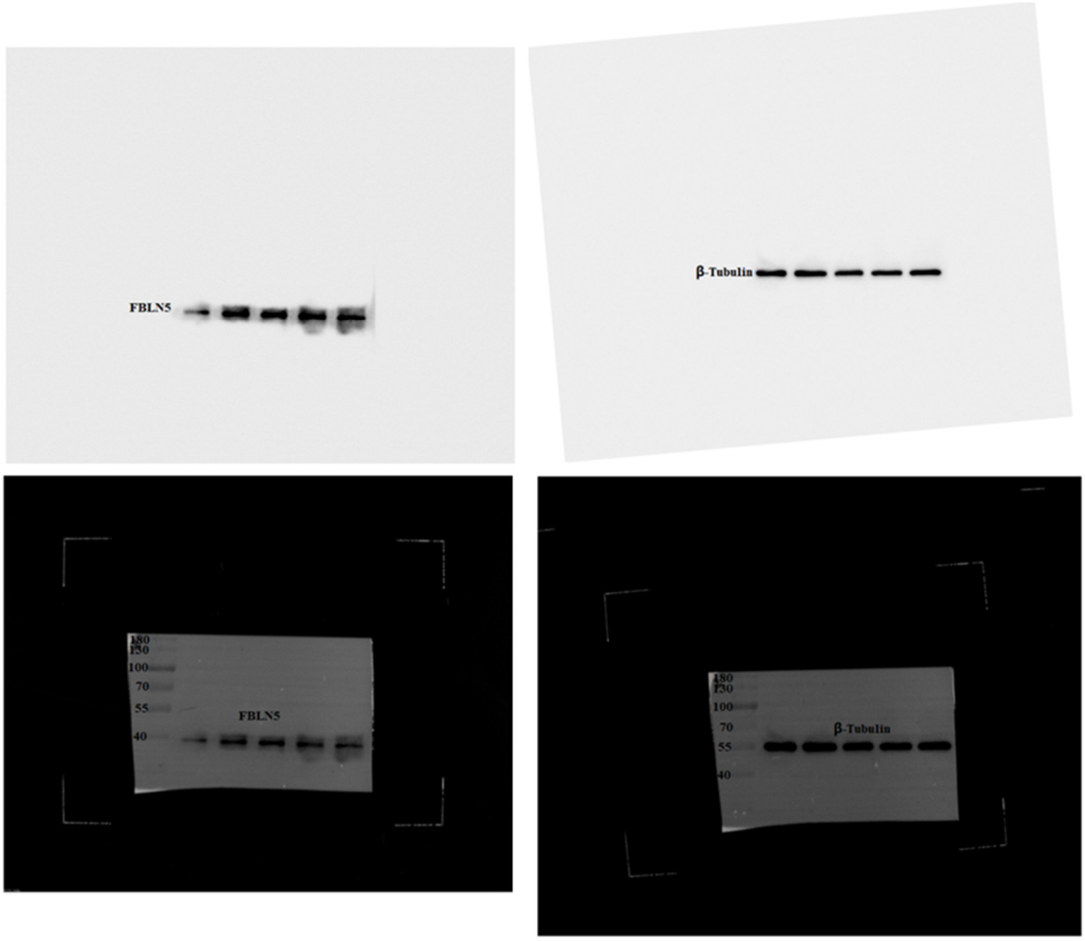

Figure S1. Western blot figures of FBLN5. The molecular weight range of the protein pre dye Marker we used is 10-180 kDa, which is 180 kDa, 130 kDa, 100 kDa, 70 kDa, 55 kDa, 40 kDa, 35 kDa, 25 kDa, 15 kDa and 10 kDa respectively.

Densitometry readings/intensity ratio of each band are as follow:

GES	AGS	BGC-823	HGC-27	MKN-28
0.546245597	1.173976061	1.308010586	1.585782484	1.276616819
0.737717207	1.656891701	1.354611092	1.427532515	1.940585495
0.929335259	1.575110203	2.057093426	1.074953206	1.308508457

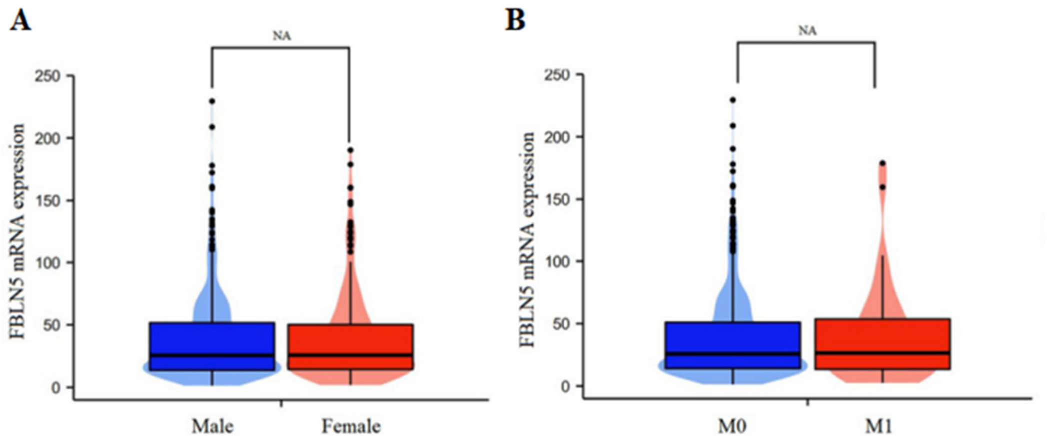

Figure S2. Correlation analysis of the expression level of FBLN5 mRNA with sex and M stage. (A) Correlation analysis between FBLN5 mRNA expression levels and sex (P>0.05) (B) Correlation analysis between FBLN5 mRNA expression levels and M stage (P>0.05).

Table S1. Baseline patient clinicopathologic characteristics.

Characteristics	Low FBLN5 expression	High FBLN5 expression	P value
n	322	322	
T stage, n (%)			< 0.001
T2	66 (10.4%)	39 (6.1%)	
T1	30 (4.7%)	9 (1.4%)	
T4	68 (10.7%)	86 (13.5%)	
T3	157 (24.7%)	181 (28.5%)	
N stage, n (%)			< 0.001
N0	104 (16.6%)	72 (11.5%)	
N1	69 (11%)	58 (9.3%)	
N3	80 (12.8%)	124 (19.8%)	
N2	62 (9.9%)	57 (9.1%)	
M stage, n (%)			0.9657
M0	289 (46.3%)	293 (47%)	
M1	21 (3.4%)	21 (3.4%)	
pTNM stage, n (%)			< 0.001
Stage1	66 (10.6%)	22 (3.5%)	
Stage4	29 (4.7%)	26 (4.2%)	
Stage3	138 (22.2%)	179 (28.8%)	
Stage2	82 (13.2%)	79 (12.7%)	
Gender, n (%)			0.8053
Male	208 (32.3%)	205 (31.8%)	
Female	114 (17.7%)	117 (18.2%)	
Age, median (IQR)	65 (56, 71)	62 (53, 69)	< 0.05
